# Understanding MOOC discontinuous usage intention: the roles of overload, fatigue, and dissatisfaction

**DOI:** 10.3389/fpsyg.2026.1728152

**Published:** 2026-09-23

**Authors:** Ting Lu, Chengyi Le

**Affiliations:** 1 School of Marxism Studies, Ningbo University, Ningbo, China; 2 Merchants' Guild Economics and Cultural Intelligent Computing Laboratory, Ningbo University, Ningbo, China; 3 School of Business, Ningbo University, Ningbo, China

**Keywords:** cognitive overload, discontinuous usage intention, massive open online courses (MOOCs), stressor-strain-outcome framework, usage fatigue

## Abstract

This study examines the links between information overload, system feature overload, social overload, and expectation disconfirmation with fatigue, dissatisfaction, and discontinuous usage among MOOC learners. We use survey data from Chinese university students, applying the Stressor-Strain-Outcome (SSO) model, cognitive overload theory, and expectation-disconfirmation theory, and test how overload and unmet expectations are associated with emotional burdens, learning experiences, and the intention to disengage. The results indicate that all three overload dimensions were positively associated with usage fatigue. Usage fatigue was also positively associated with dissatisfaction within the specified model. Gender moderated the dissatisfaction–discontinuous usage intention association, which was somewhat stronger among female participants in this sample. This study contributes to research on overload and emotional burden in educational MOOCs. Findings provide theoretical and practical implications for MOOC platform design and learner support.

## 1 Introduction

Massive Open Online Courses (MOOCs) offer flexible learning opportunities and have become an important component of the digital transformation of higher education (Kaplan and Haenlein, 2016), yet extreme attrition rates—often exceeding 90%—remain a critical educational bottleneck (Jordan, 2015; Rahmani et al., 2024). Recent dropout research has made strides using machine learning to predict early disengagement (Mikić et al., 2026; Yu et al., 2026). However, these algorithmic approaches primarily address when learners quit, leaving the psychological factors explaining why withdrawal intentions occur largely underexplored. There remains a theoretical gap in understanding how negative digital experiences—specifically those related to resource depletion and unmet expectations—are associated with emotional exhaustion and deliberate disengagement intention.

While digital overload and fatigue are well-documented in voluntary social networking sites (SNS) (Maier et al., 2015), MOOCs impose mandatory cognitive and social demands, such as graded discussion forums and peer-reviewed assignments (Fu et al., 2020). The presence of mandatory or assessed activities distinguishes MOOCs from many voluntary social networking contexts and provides a context in which overload-related experiences may warrant separate examination. Furthermore, while prior studies note demographic variations in online persistence (Jiang et al., 2018), how structural differences like gender moderate the translation of these stressors into discontinuous usage intention remains unclear.

To address these gaps, this study integrates the Stressor-Strain-Outcome (SSO) framework and Expectation-Disconfirmation Theory (EDT) into a dual-pathway model. We investigate how specific stressors (information, system feature, and social overload) and expectation disconfirmation are examined in relation to usage fatigue, dissatisfaction, and discontinuous usage intention. By incorporating gender as a contextual moderator in the dissatisfaction-disengagement link, this research delineates the psychological boundaries of MOOC discontinuous usage intention, offering evidence-based insights for targeted educational interventions.

## 2 Literature review

### 2.1 Discontinuous usage in MOOCs

Discontinuous usage refers to learners' intentional reduction, suspension, or cessation of interaction with a digital platform after initial adoption (Maier et al., 2015). In the broader MOOC literature, discontinuation may refer to reduced engagement, course abandonment, or non-completion. In the present study, however, discontinuation is operationalized as self-reported discontinuous usage intention rather than observed course withdrawal. While research has explored the drivers of continued usage—such as perceived usefulness (Li et al., 2022), satisfaction, and interactivity (Li et al., 2021)—few studies have addressed the psychological and emotional barriers to engagement. This gap emphasizes the need to move beyond simple retention metrics toward understanding the psychological costs of learning. This study employs the Stressor-Strain-Outcome (SSO) model and cognitive overload theory to investigate the relationship between overload stressors, unmet expectations, fatigue, dissatisfaction, and discontinuous usage intention. Unlike traditional educational platforms, MOOCs often require substantial learner self-regulation and sustained engagement, which makes learner fatigue and disengagement intention important topics for investigation. These contextual differences distinguish MOOCs from many SNS settings and provide a rationale for examining overload experiences within the specific context of online learning. By integrating the SSO model with expectation-disconfirmation theory (EDT), this study clarifies how strain from overload and unmet expectations relates to disengagement intention in MOOCs.

### 2.2 Stressor-strain-outcome (SSO) model in digital learning contexts

The Stressor-Strain-Outcome (SSO) model (Koeske and Koeske, 1993) provides the foundational framework for examining how environmental demands are related to psychological strain and downstream outcomes. In the context of MOOCs, the focal stressors examined in this study are information overload, system feature overload, and social overload, while usage fatigue represents the focal strain-related construct and discontinuous usage intention represents the outcome construct. Within this framework, we examine the statistical associations among these constructs rather than assume temporal or causal ordering. Cognitive load theory further provides a theoretical basis for considering how demanding digital learning environments may be related to learners' fatigue experiences (Sweller, 2010).

The SSO model has been widely applied in digital contexts, including social media, where previous research has reported associations between overload, fatigue, and disengagement (Fu et al., 2020; Zhang et al., 2016). However, applying the SSO framework to MOOCs presents a significant theoretical challenge, as educational settings differ fundamentally from voluntary social media use. To bridge this gap, recent literature has begun focusing on developing context-specific indicators for online course overload and mental fatigue (Bayne and Inan, 2022). Furthermore, recent evidence suggests that demanding coursework is associated with higher levels of mental fatigue in online learning environments (Inan et al., 2025), which has also been associated with academic resilience and digital exhaustion (An et al., 2025). Building on this trajectory, the three defined overload stressors, in this context are: (1) Information Overload: refers to learners' perceptions that the volume and complexity of learning-related information place excessive demands on their information-processing resources (Chen et al., 2011). (2) System-Feature overload: Refers to the complexity of course features, including dashboard interfaces, notifications, and interaction tools, which increase cognitive load and reduce user satisfaction (Karr-Wisniewski and Ying, 2010). (3) Social Overload: Occurs when the demand for peer interaction and collaborative tasks becomes overwhelming, which can be associated with emotional exhaustion, particularly when tied to course grading (Zhang et al., 2022). The three primary overload constructs—Information Overload, System Feature Overload, and Social Overload—are conceptualized as “External Stressors” within this framework, representing environmental demands originating external to the learner (see Figure 1).

**Figure 1:**
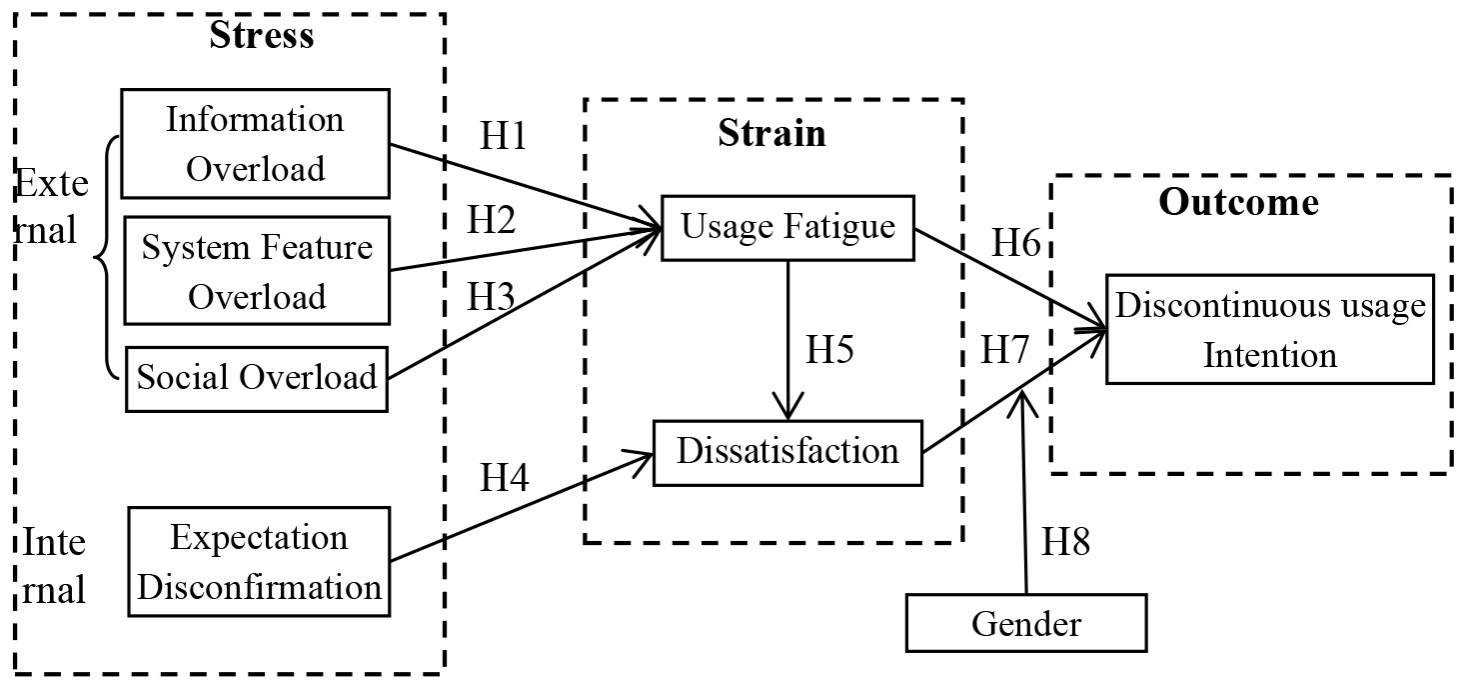
The theoretical model.

The SSO model has not been sufficiently applied to MOOCs, particularly in relation to how overload stressors and expectation disconfirmation are linked to learner fatigue, dissatisfaction, and disengagement intention. Most of the literature on the SSO model has focused on SNS and mobile learning contexts, where the role of course structure and graded tasks is less pronounced. MOOCs, by contrast, often include assessed or required learning activities that provide a distinctive context for examining learners' experiences of overload and fatigue.

While the SSO framework provides a useful perspective for examining the relationship between external digital demands and learner strain, it places less emphasis on learners' evaluations of whether their learning experiences meet prior expectations. Expectation Disconfirmation Theory (EDT) therefore provides a complementary theoretical lens for examining unmet expectations and dissatisfaction. Combining these perspectives allows the present study to examine associations involving both strain-related experiences and evaluative responses within a common MOOC framework.

### 2.3 Expectation disconfirmation and dissatisfaction

Expectation Disconfirmation Theory posits that users form expectations about a system before using it, and upon experiencing the system, they compare their expectations with the actual performance (Oliver, 1980). If the system's performance falls short of expectations, negative disconfirmation is expected to be associated with dissatisfaction, which may in turn be associated with discontinuance intention. In the context of MOOCs, learners typically enter the course with expectations about content quality, instructor interaction, and peer support. When the course fails to meet these expectations—due to excessive workload, lack of feedback, or low-quality materials—dissatisfaction arises, which is linked to disengagement intention (Gu et al., 2021). Importantly, this cognitive appraisal process remains highly relevant even in contemporary technology-enhanced environments. Recent conceptualizations suggest that learner satisfaction continues to be fundamentally shaped by the dynamic interplay between technological affordances (e.g., generative AI support) and the perceived quality of the learning experience (Lv et al., 2025).

Recent studies have also highlighted that expectation disconfirmation plays a significant role in learners' satisfaction and intention to continue (Le and Chen, 2022). In the MOOC context, prior research has applied expectation-confirmation models to explain students' continuance learning intentions and has emphasized the importance of learners' evaluations of their learning experiences (Dai et al., 2020; Rekha et al., 2023). Related research in other digital technology settings has likewise demonstrated the relevance of expectation disconfirmation to satisfaction and continued technology use (Ha et al., 2021). More broadly, these findings suggest that learners' evaluations of whether actual experiences meet prior expectations remain relevant to subsequent usage-related responses. Although expectation disconfirmation has been widely examined in online learning and digital technology contexts, its role in explaining discontinuous usage intention in MOOCs warrants further investigation.

### 2.4 Social overload and usage fatigue

Social overload refers to the mental and emotional strain associated with excessive peer interaction and social tasks, such as online discussions, group work, or peer feedback, that are often required in MOOCs. In SNS platforms, social overload occurs when users are overwhelmed by the volume of social interactions and notifications (Zhang et al., 2016). Similarly, in MOOCs, the need to engage in frequent discussions, respond to peer comments, and collaborate on group assignments can be linked to emotional exhaustion, particularly when these interactions are tied to course assessments. In traditional SNS platforms, social overload is typically voluntary, with users engaging or disengaging as they wish.

In some MOOCs, social interactions may be required or linked to academic evaluation, making course-related peer interaction a potentially important form of social demand. Peer review is one common form of assessed peer interaction in MOOCs and may contribute to learners' perceived social demands (Suen, 2014). This contextual feature makes social overload theoretically relevant to the study of learner fatigue in MOOCs. The present study therefore examines whether social overload is positively associated with usage fatigue in this educational context. The role of social overload in MOOCs has not been fully explored. While social overload is well-documented in SNS contexts, where user participation is voluntary, its association in MOOCs, where social interactions are often tied to course requirements and grading, may differ significantly. The role of course-related social demands in MOOC fatigue warrants further empirical examination. The study of overload beyond social media supports that information and social demands are critical in explaining discontinuance intentions in digital spaces (Chung et al., 2023).

### 2.5 Gender differences in overload and discontinuous usage intention

Gender differences in emotional regulation, social sensitivity, and media engagement have been well-documented in the literature (Gross, 1998; Bright et al., 2016). Prior research has documented gender-related variation in online learning participation, engagement, and persistence, although the reported patterns are heterogeneous across contexts (Jiang et al., 2018; Rizvi et al., 2024). Some studies have suggested that gender may be associated with differences in learners' experiences of online interaction, evaluation, and engagement. However, the psychological processes underlying such differences remain insufficiently established.

Self-Determination Theory (SDT) provides one possible theoretical perspective for considering individual differences in responses to learning environments, particularly with respect to autonomy, competence, and relatedness (Deci and Ryan, 2000). Nevertheless, the present study does not directly assess these psychological needs, gender-role orientations, sensitivity to social evaluation, or tolerance of unmet expectations. Accordingly, these constructs are not treated as explanatory mechanisms in the present study.

Instead, we examine gender as a contextual moderator of the association between dissatisfaction and discontinuous usage intention. This approach allows us to test whether the strength of the statistical association differs across gender groups without assuming that such differences necessarily reflect a specific psychological process. The gender hypothesis is therefore formulated as an empirical moderation hypothesis that warrants further investigation across diverse samples and with directly measured psychological mechanisms.

## 3 Research design

### 3.1 Research objectives

The primary objective of this study is to investigate the relationships between information overload, system feature overload, social overload, and expectation disconfirmation and their associations with fatigue, dissatisfaction, and discontinuous usage intention among MOOC learners. Additionally, this study explores how gender moderates the relationship between dissatisfaction and discontinuous usage intention.

### 3.2 Hypotheses development

#### 3.2.1 Information Overload → Fatigue

The large volume of content in MOOCs, including extensive lectures, readings, and assignments, combined with frequent notifications, can relate to fatigue, according to the SSO model and cognitive load theory. Unlike casual SNS use, where users can easily opt out, MOOCs involve mandatory tasks and graded components, making the cognitive load more significant. This task sequencing and scoring constraint mean the cost of skipping or delaying is higher, acting as a continuous source of sustained cognitive strain and fatigue. Therefore, we hypothesize that:

**H1:** Information overload is positively associated with usage fatigue in MOOCs.

#### 3.2.2 System Feature Overload → Fatigue

The complexity and abundance of MOOC system features, when overwhelming to the learners, are associated with increased cognitive load and are positively associated with fatigue (Fu et al., 2020). Given that in MOOCs, these features are often tied to assessment and progress tracking, learners may experience more strain. The function-bundled course dashboards (e.g., combining grades, assignments, notifications, and badges) increase the learners' extrinsic cognitive load and task-switching costs, which are positively correlated with higher fatigue. Thus, we hypothesize:

**H2:** System feature overload is positively associated with usage fatigue in MOOCs.

#### 3.2.3 Social Overload → Fatigue

Social overload may arise from demanding peer interactions, including graded discussions, peer reviews, and other course-related social tasks. Such peer engagement can be associated with emotional exhaustion and cognitive overload, especially when learners feel pressured to respond to or engage with their peers (Zhang et al., 2022). Because discussions and peer reviews may be tied to academic requirements in some MOOCs, peer interaction represents a theoretically relevant source of learner strain. Consistent with the SSO framework, we therefore expect social overload to be positively associated with usage fatigue. Therefore, we hypothesize:

**H3:** Social overload is positively associated with usage fatigue in MOOCs.

#### 3.2.4 Expectation Disconfirmation → Dissatisfaction

This hypothesis is based on the Expectation-Disconfirmation Theory (Oliver, 1980; Bhattacherjee, 2001), which suggests that unmet expectations are associated with negative emotional responses, including dissatisfaction. When the actual learning experience does not meet the learner's expectations regarding course quality, instructor engagement, or content relevance, negative disconfirmation is expected to be associated with dissatisfaction (Gu et al., 2021). Hence, we hypothesize:

**H4:** Expectation disconfirmation is positively associated with dissatisfaction in MOOCs.

#### 3.2.5 Usage Fatigue → Dissatisfaction

Usage fatigue and dissatisfaction are conceptually related but represent distinct constructs in this study. Usage fatigue reflects learners' perceived mental and emotional exhaustion during MOOC use, whereas dissatisfaction reflects a negative evaluation of the learning experience. Based on the SSO framework and prior research linking fatigue-related experiences with negative evaluations of digital services, we examine whether usage fatigue is positively associated with dissatisfaction. Given the cross-sectional design, this hypothesis concerns the statistical association between the two constructs rather than temporal precedence. Therefore, we hypothesize:

**H5:** Usage fatigue is positively associated with dissatisfaction in MOOCs.

#### 3.2.6 Usage Fatigue → Discontinuous

Fatigued learners are less likely to continue their engagement with MOOCs, especially when they feel the mental and emotional strain exceeds the value of continuing (Hone and El Said, 2016). Prior research suggests that fatigue-related experiences are associated with lower intentions to continue using digital services. In the present study, we therefore examine whether usage fatigue is positively associated with discontinuous usage intention among MOOC learners. Therefore, we hypothesize:

**H6:** Usage fatigue is positively associated with discontinuous usage intention in MOOCs.

#### 3.2.7 Dissatisfaction → discontinuous

Usage Intention Dissatisfaction is associated with discontinuous usage intention in digital platforms (Gu et al., 2021; Tan et al., 2023). In MOOCs, when learners report dissatisfaction in conjunction with unmet expectations or higher levels of usage fatigue, they may report stronger discontinuous usage intentions. This hypothesis builds on the Expectation-Disconfirmation Theory (Bhattacherjee, 2001), which links dissatisfaction to discontinuous usage intention. Therefore, we hypothesize:

**H7:** Dissatisfaction is positively associated with discontinuous usage intention in MOOCs.

#### 3.2.8 Gender Moderation: Dissatisfaction → Discontinuous Usage Intention

Research on online learning has noted that male and female learners often exhibit comparable responses to structural demands (e.g., information volume, system complexity), whereas differences emerge more consistently at the stage of emotional evaluation and behavioral intention (Jiang et al., 2018). Self-Determination Theory offers a plausible interpretive backdrop for this pattern, as prior literature suggests that social relatedness and feedback sensitivity may carry differential motivational relevance across gender groups in educational settings (Shao and Chen, 2021). Building on this perspective, we propose that gender may moderate the association between dissatisfaction and discontinuous usage intention, with a stronger association expected among female learners. SDT is used here as a theoretical lens for hypothesis development rather than as an empirically tested explanation, because the underlying psychological needs were not directly measured. Therefore, we hypothesize:

**H8:** Gender moderates the relationship between dissatisfaction and discontinuous usage intention, with females showing a stronger association.

Based on the hypothesized relationships of the core variables, this study constructs a research model (see Figure 1).

### 3.3 Variables and measurement

All constructs in this study were measured using previously validated scales, adapted to the MOOC context, and translated into Chinese through a standard back-translation process. Items were rated on a 7-point Likert scale ranging from 1 (strongly disagree) to 7 (strongly agree). The following constructs were measured in this study:

Information Overload: Adapted from the scale developed by Chen et al. (2011), measuring the degree to which learners feel overwhelmed by the volume and complexity of course materials and notifications.

System Feature Overload: Based on Karr-Wisniewski and Ying (2010), assessing the complexity and overwhelming nature of system features, including dashboards, notifications, and peer interactions.

Social Overload: Based on Zhang et al. (2016), measuring the emotional strain associated with excessive peer interaction demands and group assignments.

Expectation Disconfirmation: Adapted from Bhattacherjee (2001), measuring the gap between learners' initial expectations and their actual learning experience.

Usage Fatigue: Adapted from established fatigue measures and operationalized in prior discontinuance research (Ravindran et al., 2014; Zhang et al., 2016), which assesses emotional exhaustion and cognitive fatigue in the context of digital platform use.

Dissatisfaction: Measured with items from Gu et al. (2021), assessing learners' dissatisfaction with their course experience.

Discontinuous Usage Intention: Adapted from established measures of discontinuance intention (Maier et al., 2015), assessing learners' intention to reduce, temporarily suspend, switch from, or stop using the MOOC.

### 3.4 Sample selection

Data were collected through an online questionnaire distributed via popular Chinese social media platforms, WeChat and QQ. The selection of these specific platforms is justified by the target population: in the Chinese higher education context, WeChat and QQ function as the primary digital infrastructures for university academic groups, class administration, and peer communication. Consequently, distributing the survey within these networks provided direct access to active student cohorts engaging in online learning.

To mitigate the inherent self-selection bias associated with snowball sampling, the survey employed specific screening questions to filter out non-target users, ensuring only individuals who had taken at least one MOOC within the past 6 months were included. Participation was voluntary and anonymous. Furthermore, responses with completion times (< 30 s) or overly uniform answering patterns were excluded to maintain data integrity. The data collection yielded a total of 427 initial responses. Following the data cleaning process, a final sample size of 403 participants was retained for the analysis.

## 4 Results

### 4.1 Descriptive statistics

A total of 427 questionnaires were collected, resulting in 403 valid samples after data cleaning (yielding a valid-response rate of 94.4%). The respondents were predominantly young adults, with 78.9% aged 19–24, 14.1% aged 25–30, and the remaining 6.9% in other age groups. Females slightly outnumbered males (53.6% vs. 46.4%). In terms of education, 69.5% were undergraduates, 18.9% college students, and 11.7% graduate students. Weekly MOOC usage varied: 30.3% used the platform for ≤ 2 h, 38.7% used it for 2–4 h, 20.6% used it for 4–6 h, and 10.4% used it for >6 h. The sample was predominantly composed of young university students, which reflects the recruitment context of the present study. Because participants were recruited through non-probability convenience and snowball sampling, the sample should not be regarded as statistically representative of the broader MOOC learner population. The demographic similarities to prior studies do not equate to population-level representativeness, and generalizations from this specific cohort to the broader online-learning population must be approached with strict caution, potentially over representing digitally active and engaged learners.

### 4.2 Reliability and validity analysis

We assessed the psychometric properties of each construct using SPSS 26.0 and AMOS 26.0. Internal consistency: Cronbach's α for all scales exceeded the commonly used 0.80 threshold (Table 1). In general, an α above 0.8 is considered evidence of strong reliability, indicating that our multi-item scales consistently measure their intended constructs.

#### 4.2.1 Convergent validity

Confirmatory factor analysis showed that all standardized factor loadings were high, and each variable's composite reliability (CR) exceeded 0.90. Crucially, the Average Variance Extracted (AVE) for every construct was above 0.5 (see Table 1). An AVE ≥ 0.50 is the standard criterion for acceptable convergent validity, meaning each latent variable explains over half of the variance in its items. The high internal consistency primarily reflects the narrow semantic focus of adapting established scales exclusively to the MOOC context.

**Table 1:** Cronbach's α and AVE.

Variable	Items	Standard loading	Cronbach's α	CR	AVE
Information overload (IO)	IO 1	0.948	0.943	0.943	0.807
	IO 2	0.892			
	IO 3	0.887			
	IO 4	0.863			
System feature overload (SFO)	SFO 1	0.962	0.947	0.947	0.818
	SFO 2	0.871			
	SFO 3	0.907			
	SFO 4	0.875			
Social overload (SO)	SO 1	0.955	0.935	0.936	0.829
	SO 2	0.886			
	SO 3	0.888			
Expectation disconfirmation (ED)	ED 1	0.933	0.936	0.936	0.830
	ED 2	0.897			
	ED 3	0.903			
Usage fatigue (UF)	UF 1	0.950	0.937	0.938	0.790
	UF 2	0.865			
	UF 3	0.874			
	UF 4	0.863			
Dissatisfaction (DIS)	DIS 1	0.948	0.948	0.948	0.822
	DIS 2	0.893			
	DIS 3	0.889			
	DIS 4	0.895			
discontinuous usage intention (DUI)	DUI 1	0.944	0.937	0.937	0.790
	DUI 2	0.869			
	DUI 3	0.845			
	DUI 4	0.893			

As detailed in the subsequent discriminant validity analysis, the subsequent discriminant-validity analyses provide evidence consistent with empirical distinctiveness among the constructs.

While the high internal consistency indicates potential intra-construct item redundancy driven by the narrow semantic focus of MOOC-specific measures, these findings do not by themselves establish full conceptual distinctiveness among the constructs. The Fornell–Larcker results were consistent with discriminant validity, although this criterion does not by itself establish conceptual independence among the constructs.

#### 4.2.2 Discriminant validity

While the exceptionally high internal consistency and AVE reflect strong psychometric reliability, we acknowledge that they may also indicate semantic redundancy among the measurement items, driven by the highly specific context of mandatory MOOCs. To ensure that this narrow semantic focus did not compromise construct distinctiveness, we strictly applied the Fornell-Larcker criterion. As shown in Table 2, the square root of the AVE for each construct substantially exceeded all inter-construct correlations. The Fornell–Larcker results provide one source of evidence regarding discriminant validity, but should be interpreted alongside other evidence, particularly given the relatively high inter-construct correlations.

**Table 2:** AVE square root and correlation coefficient.

	ED	SO	SFO	IO	UF	DIS	DUI
ED	**0.911**						
SO	0.740	**0.910**					
SFO	0.760	0.728	**0.905**				
IO	0.709	0.724	0.738	**0.898**			
UF	0.664	0.737	0.717	0.718	**0.889**		
DIS	0.726	0.735	0.712	0.706	0.717	**0.906**	
DUI	0.622	0.671	0.652	0.656	0.734	0.768	**0.889**

Bold values represent the square roots of the average variance extracted (AVE) for the corresponding constructs.

While adherence to the Fornell-Larcker criterion provides necessary evidence compatible with discriminant validity, it does not definitively establish conceptual independence, particularly given the high inter-construct correlations observed. Therefore, we further calculated the Heterotrait-Monotrait (HTMT) ratio of correlations for all construct pairs.

As presented in Table 3, all HTMT values are strictly below the conservative threshold of 0.90 advocated by Henseler et al. (2015), with bootstrap 95% confidence intervals (1,000 samples) that do not include 1.0. The highest HTMT value was observed for the DIS–DUI pair [0.815, 95% CI (0.772, 0.855)], followed by SFO–ED [0.807, 95% CI (0.768, 0.844)] and SO–ED [0.791, 95% CI (0.747, 0.833)]. Because Fornell–Larcker and HTMT are diagnostic criteria rather than definitive proof of discriminant validity, we interpret these results as supporting the empirical distinctiveness of the constructs within this sample, rather than as definitive proof of absolute conceptual independence. Finally, the complete measurement items are provided in Table A1 to allow readers to transparently assess potential semantic overlap introduced during the adaptation of these scales to the MOOC context.

**Table 3:** HTMT (heterotrait-monotrait ratio) matrix.

	ED	SO	SFO	IO	UF	DIS	DUI
ED	—						
SO	0.791	—					
SFO	0.807	0.774	—				
IO	0.754	0.770	0.780	—			
UF	0.709	0.788	0.761	0.763	—		
DIS	0.771	0.780	0.752	0.745	0.761	—	
DUI	0.664	0.717	0.692	0.697	0.783	0.815	—

### 4.3 Common method bias test

Because all principal constructs were measured using self-reported responses collected in a single survey, common-method variance is a potential source of bias. Several procedural remedies were applied, including assuring anonymity, emphasizing that there were no right or wrong answers, and encouraging honest responses. We also conducted a Common Latent Factor (CLF) analysis by introducing an unmeasured latent factor across the observed indicators. The inclusion of the CLF did not materially alter the structural path estimates, and model fit did not improve meaningfully (ΔCFI < 0.01). These diagnostics did not indicate that common-method variance alone accounted for the observed relationships. Nevertheless, statistical remedies cannot eliminate common-method variance, and the single-source, cross-sectional design remains an important limitation of the study.

### 4.4 Structural model fit and path analysis

We evaluated the overall structural model fit using AMOS 26.0. The model demonstrated good fit to the empirical data, yielding the following indices: χ^2^/df = 1.85 (< 3.0), CFI = 0.985 (≥ 0.95), TLI = 0.980 (≥ 0.95), RMSEA = 0.042 ( ≤ 0.08), and SRMR = 0.038 ( ≤ 0.08). According to established structural equation modeling guidelines, these indices indicate good fit between the specified model and the observed covariance structure (Figure 1). However, we emphasize that good model fit merely indicates that the hypothesized covariance structure is compatible with the observed data; it does not establish that the proposed directional sequence actually occurred over time, nor does it exclude plausible alternative directional models.

We then examined each hypothesized path (see Table 4). Key results were:

**Table 4:** Coefficient estimation results and hypothesis testing.

Hypotheses	Path	Estimate (β)	S.E.	*t*-value	*P*-value	95% CI	Status
	IO → UF	0.288^***^	0.048	5.989	< 0.001	[0.194, 0.383]	Supported
H2	SFO → UF	0.284^***^	0.049	5.807	< 0.001	[0.188, 0.380]	Supported
H3	SO → UF	0.311^***^	0.049	6.391	< 0.001	[0.215, 0.406]	Supported
H4	ED → DIS	0.427^***^	0.042	10.182	< 0.001	[0.345, 0.510]	Supported
H5	UF → DIS	0.419^***^	0.042	10.001	< 0.001	[0.337, 0.502]	Supported
H6	UF → DUI	0.363^***^	0.042	8.592	< 0.001	[0.280, 0.446]	Supported
H7	DIS → DUI	0.499^***^	0.042	11.818	< 0.001	[0.416, 0.582]	Supported

^***^p < 0.001.

#### 4.4.1 Stressors and Strain (H1, H2, H3):

Consistent with the SSO model, all three external overload stressors were positively associated with usage fatigue. Information Overload was positively associated with usage fatigue [β = 0.288, *p* < 0.001, 95% CI (0.194, 0.383)], supporting H1. System Feature Overload was positively associated with usage fatigue [β = 0.284, *p* < 0.001, 95% CI (0.188, 0.380)], supporting H2. Notably, Social Overload was positively associated with usage fatigue [β = 0.311, *p* < 0.001, 95% CI (0.215, 0.406)], supporting H3. Although this was the numerically largest standardized coefficient among the three overload dimensions, the differences were small (Δβ ≤ 0.027), and no formal coefficient-equality test was conducted. Therefore, the three coefficients should not be interpreted as statistically different in magnitude.

#### 4.4.2 Strain, Expectations, and Dissatisfaction (H4, H5):

The expectation disconfirmation pathway (EDT) and the SSO pathway converged on dissatisfaction. Expectation Disconfirmation (ED → DIS) had a significant positive association [β = 0.427, *p* < 0.001, 95% CI (0.345, 0.510)], supporting H4. Furthermore, usage fatigue (UF → DIS) was positively associated with dissatisfaction [β = 0.419, *p* < 0.001, 95% CI (0.337, 0.502)], supporting H5. This structural convergence yields evidence that lower learner satisfaction is jointly associated with perceived resource depletion (fatigue) and the cognitive gap between initial expectation and actual reality.

#### 4.4.3 Discontinuous usage Intention (H6, H7):

Both variables showed significant positive associations with discontinuous usage intention within the specified SEM. Usage fatigue showed a significant positive association with discontinuous usage intention [β = 0.363, *p* < 0.001, 95% CI (0.280, 0.446)], supporting H6. Dissatisfaction showed the largest standardized coefficient among the modeled paths to discontinuous usage intention [β = 0.499, *p* < 0.001, 95% CI (0.416, 0.582)], supporting H7. These estimates indicate statistically significant positive associations within the specified model.

### 4.5 Mediation analysis

We tested the indirect effects of the key pathways using bias-corrected bootstrapping (2,000 samples, 95% confidence intervals). Specifically, we tested five mediation paths: three paths mediated by usage fatigue (IO → UF → DIS, SFO → UF → DIS, SO → UF → DIS), and two paths mediated by Dissatisfaction (UF → DIS → DUI, ED → DIS → DUI). The results (Table 5) show that all five hypothesized indirect effects were statistically significant, as the 95% confidence intervals did not include zero.

**Table 5:** Indirect effect test.

Path	Indirect effect	Standard error	Boot LLCI	Boot ULCI	*P*-value
IO → UF → DIS	0.121^***^	0.024	0.077	0.173	< 0.001
SFO → UF → DIS	0.119^***^	0.024	0.076	0.17	< 0.001
SO → UF → DIS	0.131^***^	0.025	0.087	0.181	< 0.001
UF → DIS → DUI	0.209^***^	0.026	0.162	0.261	< 0.001
ED → DIS → DUI	0.212^***^	0.027	0.161	0.267	< 0.001

^***^p < 0.001.

These findings provide statistical support for the specified indirect pathways, but do not establish temporal mediation or causal mechanisms.

### 4.6 Moderating effect analysis

We examined whether gender moderates the association between dissatisfaction (DIS) and discontinuous usage intention (DUI) (H8). While covariance-based SEM was employed to evaluate the primary mediation pathways, integrating latent interaction terms often introduces significant non-normality and convergence complexities. Therefore, we utilized Hierarchical Regression Analysis for the moderation test (McClelland and Judd, 1993; Aguinis et al., 2005). HRA provides statistical support for testing categorical moderators and offers a transparent estimation of the incremental explained variance (ΔR^2^), ensuring methodological clarity without overcomplicating the structural model. Gender was dummy-coded (0 = male, 1 = female).

#### 4.6.1 Hierarchical Regression Results

As reported in Table 6, we estimated three sequential models: Model 1 (baseline): DUI regressed on DIS only. The model explained 56.4% of variance (β = 0.751, *p* < 0.001; *R*^2^ = 0.564) in discontinuous usage intention. Model 2 (+ gender): Adding gender significantly improved the explanatory power (R^2^ = 0.580). The main effect of gender was significant (β = −0.124, *p* < 0.001), while the association between dissatisfaction and discontinuous usage intention remained statistically significant (β = 0.743, *p* < 0.001). Model 3 (+ interaction): Introducing the interaction DIS × Gender significantly improved model fit (ΔR^2^ = 0.008, *p* < 0.01). The interaction coefficient was positive and significant (β = 0.090, *p* < 0.01), supporting H8. The interaction explained a small additional proportion of variance (Δ*R*^2^ = 0.008). We therefore interpret the moderation effect as statistically detectable but modest in magnitude.

**Table 6:** Test of the moderation effect.

Independent variable	Dependent variable: discontinuous usage intention		
	Model 1	Model 2	Model 3
Dissatisfaction	0.751^***^	0.743^***^	0.745^***^
Gender		−0.124^***^	−0.125^***^
Dissatisfaction ^*^ gender			0.090 ^**^
*R^2^*	0.564	0.580	0.588
*F*	519.251^***^	275.815^***^	189.634^***^

^***^p < 0.001, ^**^p < 0.01.

#### 4.6.2 Subgroup Analysis and Chow Test

To further examine the pattern of the interaction, we applied overall-sample z-standardization to DUI, DIS, and UF, subsequently estimating the identical structural model (DUI ~ UF + DIS) within each gender subgroup. The structural path from dissatisfaction to discontinuous usage intention was notably steeper for female learners [β = 0.531, 95% CI (0.418, 0.644), *p* < 0.001] compared to male learners [β = 0.443, 95% CI (0.327, 0.559), *p* < 0.001]. A Chow test for structural stability supported a statistically significant cross-group difference in this specific parameter (*F* = 9.340, *p* < 0.01). The subgroup estimates were consistent with the pooled interaction test and showed a stronger estimated DIS–DUI association among female learners. This pattern is consistent with H8, while the magnitude of the interaction should be interpreted as modest.

#### 4.6.3 Simple slope

The simple-slope analysis (Figure 2) illustrates the interaction pattern: the estimated slope for female learners (Gender = 1) regarding the DIS → DUI relationship is steeper than that for male learners (Gender = 0). Substantively, this indicates that at equivalent levels of dissatisfaction, the statistical association with discontinuous usage intention is modestly stronger among female learners compared to male learners in this specific sample.

**Figure 2:**
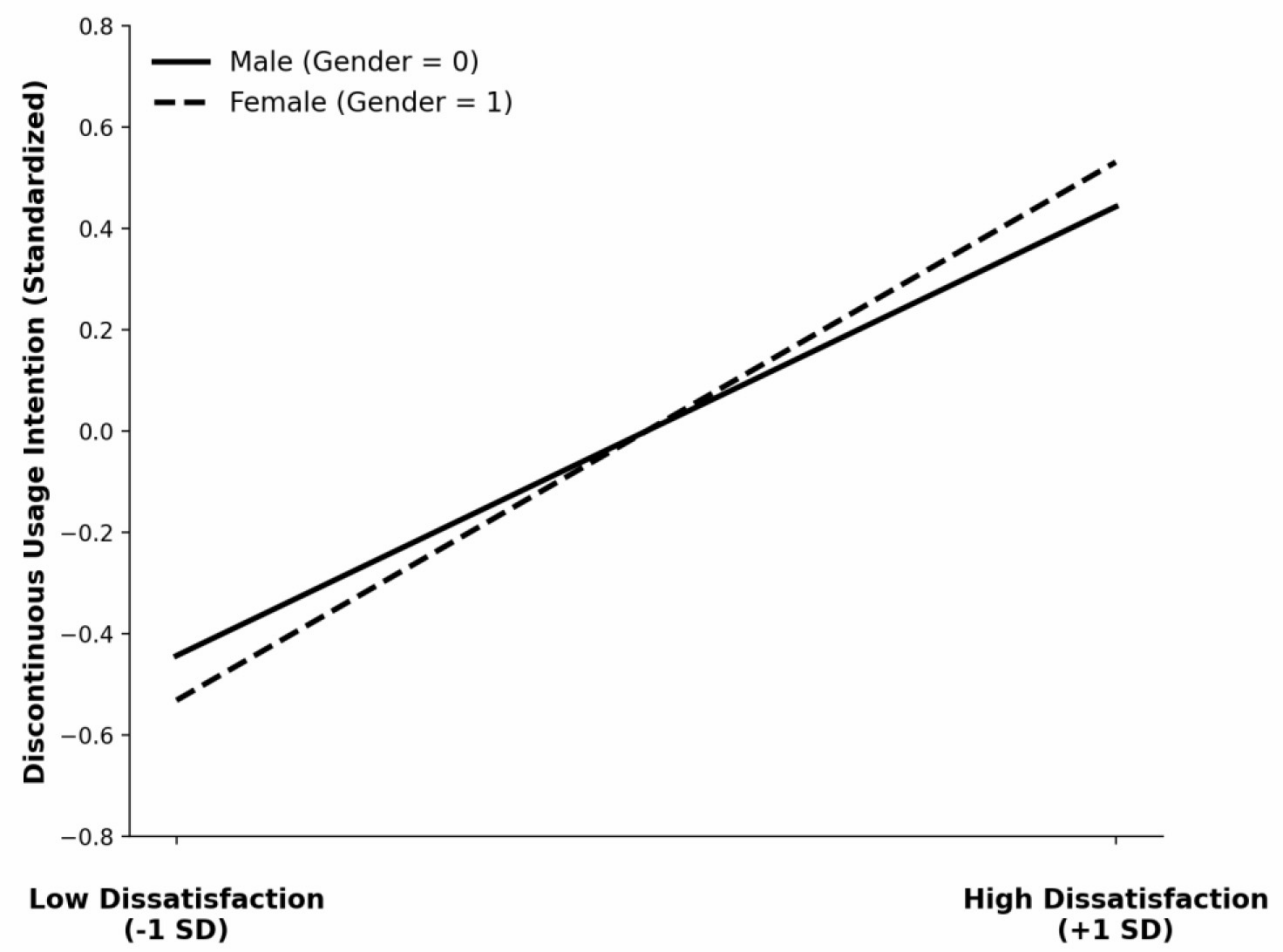
The moderating effect of gender.

## 5 Discussion and conclusion

### 5.1 Discussion

This study aimed to investigate the relationships between information overload, System Feature Overload, social overload, and expectation disconfirmation, and their associations with fatigue, dissatisfaction, and discontinuous usage intention in MOOCs, while also considering the moderating role of gender. The findings indicate that the three overload dimensions were positively associated with usage fatigue and that usage fatigue was positively associated with dissatisfaction and discontinuous usage intention within the specified model. The findings can be interpreted from two complementary theoretical perspectives: the SSO perspective on overload and fatigue, and the expectation-confirmation perspective on dissatisfaction and discontinuous usage intention.

#### 5.1.1 Capability-limitation pathway: associations between overload and fatigue

The first hypothesized pathway concerns the association between overload and usage fatigue. Consistent with the SSO perspective, the findings indicate that information overload, system feature overload, and social overload were all positively associated with usage fatigue. Social overload showed the numerically largest standardized coefficient (β = 0.311), followed by information overload (β = 0.288) and system feature overload (β = 0.284). However, the differences among these coefficients were small (Δβ ≤ 0.027), and no formal coefficient-equality test was conducted. Therefore, the results should not be interpreted as evidence that social overload has a statistically stronger association with usage fatigue than the other two overload dimensions.

The MOOC context nevertheless provides a theoretically relevant setting in which course-related peer interaction may represent a salient form of social demand, particularly when discussions or peer reviews are required or assessed. This interpretation is compatible with prior research indicating that non-optional coursework demands can be associated with learner fatigue in online educational settings (Inan et al., 2025). The present findings therefore extend the application of the SSO perspective to MOOCs by showing that information, system-feature, and social overload are each associated with usage fatigue in the sampled learners.

#### 5.1.2 Value-appraisal pathway: associations with dissatisfaction and discontinuous usage intention

The second theoretical perspective concerns the associations involving expectation disconfirmation, dissatisfaction, and discontinuous usage intention. This pathway aligns with the findings of Gu et al. (2021) and Bhattacherjee (2001), who discovered that expectation disconfirmation is positively associated with dissatisfaction in digital learning environments.

Dissatisfaction showed the largest standardized coefficient among the modeled paths to discontinuous usage intention (β = 0.499). This result indicates that dissatisfaction was strongly associated with discontinuous usage intention within the specified model. However, the coefficient should not be interpreted as evidence that dissatisfaction is a dominant behavioral determinant, particularly given the cross-sectional design. This pattern is theoretically compatible with the view that learners' evaluations of unmet expectations may be relevant to discontinuous usage intention.

#### 5.1.3 Gender moderation: variation in the dissatisfaction–discontinuation-intention association

The empirical conclusion from the subgroup and moderation analyses is that gender modestly moderated the association between dissatisfaction and discontinuous usage intention in this sample. Specifically, the statistical association was somewhat stronger among female participants (β = 0.531) compared to male participants (β = 0.443). While this effect is statistically detectable, it is small in incremental explanatory terms (Δ*R*^2^= 0.008).

Prior literature drawing on Self-Determination Theory (SDT) suggests that differences in relational sensitivity, relatedness needs, or responses to social evaluation might inform such patterns (Shao and Chen, 2021). However, gender was operationalized as a binary demographic variable (0 = male, 1 = female) in this study. We did not directly assess psychological need prioritization, gender-role orientation, tolerance of unmet expectations, or interpersonal friction. Given the small incremental variance explained, we emphasize the need to avoid constructing a broad psychological account of male vs. female MOOC behavior around this result, thereby adhering to empirical boundaries and mitigating the risk of essentializing gender differences.

### 5.2 Theoretical implications

This study provides several theoretical observations concerning the application of the SSO and expectation-confirmation perspectives to MOOC discontinuation usage intention.

#### 5.2.1 Integrating the SSO and expectation-confirmation perspectives:

Our empirical findings indicate that the observed structural associations—specifically, the significant indirect pathway from usage fatigue to discontinuous usage intention via dissatisfaction—are compatible with a sequential integration of the SSO and EDT perspectives. Building on this structural compatibility, future research could examine whether cognitive strain is associated with variation in how learners evaluate unmet expectations. This formulation provides a conceptual foundation for future longitudinal or experimental research to investigate the dynamic interplay between capacity limitation and evaluative judgments.

#### 5.2.2 Contextualizing social overload in MOOC environments:

The findings position course-related peer interaction as a theoretically relevant contextual dimension of overload in MOOCs. All three overload dimensions were positively associated with usage fatigue, with social overload showing the numerically largest standardized coefficient; however, the coefficient differences were modest and were not formally tested for statistical superiority. Accordingly, the result is best understood as evidence that social overload is a relevant correlate of learner fatigue alongside information and system feature overload, rather than as evidence of dominance. More importantly, the MOOC context introduces a distinctive form of social demand when peer discussions or assessments are required or tied to academic evaluation. This contextual feature highlights a potentially relevant boundary condition for extending the SSO framework to educational settings and suggests that the role of social demands may warrant greater attention in future research on learner fatigue in MOOCs.

#### 5.2.3 Identifying a potential boundary condition for discontinuation usage intention:

Gender significantly moderated the association between dissatisfaction and discontinuous usage intention, although the incremental variance explained by the interaction was small. This finding identifies a potential boundary condition for the association within the present sample, while the psychological processes underlying the observed difference remain to be tested.

### 5.3 Practical implications

Based on the observed associations between overload, dissatisfaction, and discontinuous usage intention, we cautiously propose several exploratory avenues for MOOC platform design. Since these interventions were not directly evaluated in the current study, they serve as hypothesized directions that warrant future experimental validation.

#### 5.3.1 Exploring interventions targeting social overload

The finding that social overload was positively associated with usage fatigue suggests that course-related peer interaction may represent a relevant form of learner demand in MOOC settings. Future platform iterations might benefit from exploring structural modifications to peer-to-peer evaluation mechanisms. For instance, subsequent applied research could utilize A/B testing to determine whether highly structured, rubric-based grading interfaces mitigate emotional exhaustion more effectively than traditional open-ended reviews (Suen, 2014). Furthermore, transitioning high-demand or assessed discussions toward incentivized, optional collaborative formats might reduce perceived social demands. Finally, future experimental designs could assess the strain-reducing efficacy of integrating algorithmic feedback templates, exploring whether such tools standardize peer communication and reduce perceived social-evaluative demands without compromising pedagogical quality.

#### 5.3.2 Exploring expectation calibration and learner dissatisfaction

Given that dissatisfaction showed a larger standardized coefficient than usage fatigue in the specified model, the findings suggest that expectation-related evaluations may warrant attention alongside fatigue-related experiences. A salient exploratory direction involves transparent expectation calibration. Future research could examine whether high-fidelity course descriptions—covering workload expectations, required peer interaction, and expected instructor feedback times—are associated with better expectation alignment and lower dissatisfaction. This direction is also consistent with recent evidence that online teacher feedback is relevant to learners' engagement through technological and motivational processes (Zheng and Pan, 2026). Additionally, evaluating the implementation of timely, personalized feedback loops (e.g., via AI-tutor systems) represents a viable future intervention. Testing such interventions may clarify whether improved expectation calibration is associated with lower dissatisfaction and discontinuous usage intention.

#### 5.3.2 Exploring gender-sensitive learning configurations

Given that the statistical association between dissatisfaction and discontinuous usage intention was modestly stronger among female learners in this sample, future platform designs might explore whether supportive, non-judgmental digital environments differentially benefit specific user demographics. Potential interventions for future testing could include flexible interface options and customizable support features that accommodate different learner preferences. These possibilities should be evaluated empirically rather than designed on the assumption that female and male learners possess inherently different psychological needs or processing styles.

### 5.4 Limitations and future research

Several boundary conditions of this study present opportunities for subsequent research. First, the reliance on cross-sectional, single-source self-report data restricts the ability to establish definitive temporal precedence and introduces inherent risks of common method variance (CMV). Although the *post-hoc* CLF analysis did not suggest that a single common method factor accounted for the observed relationships, the temporal ordering implied by the theoretical model cannot be established. Future inquiries should employ multi-wave longitudinal designs to rigorously map the temporal sequence proposed by the integrated SSO and EDT pathways.

Second, the dependent variable in this study is Discontinuous Usage Intention, which represents self-reported behavioral intention rather than actual platform dropout. While behavioral intention is a well-established theoretical precursor to actual behavior, the present results strictly explain variation in self-reported discontinuation usage intentions. They do not empirically demonstrate that the identified variables predict actual observed withdrawal. Future studies must incorporate objective behavioral tracking data—such as platform clickstreams, log-out rates, or assignment submission records—to bridge the intention-behavior gap and establish the predictive validity of the associations identified in the present model with actual course discontinuation.

Third, the use of non-probability snowball sampling via WeChat and QQ inherently limits external validity. While this strategy effectively accessed our target demographic of digitally active university cohorts, it may introduce self-selection bias and may disproportionately attract learners who are more active in digital environments. Consequently, the structural coefficients estimated in this study primarily reflect the specific covariance structure of this biased sample; they cannot be assumed to provide an unbiased representation of the true population parameters. To ascertain the broader applicability and structural stability of the dual-pathway model, subsequent studies are required to implement stratified random sampling across diverse educational institutions and sociodemographic strata.

Finally, while this study identifies a positive association between social overload and usage fatigue in the sampled MOOC context, the specific contextual triggers of this stressor warrant deeper exploration. Integrating qualitative methodologies, such as in-depth interviews or focus groups, could unpack how learners subjectively interpret peer-assessment pressure and value discrepancy. Building upon these mixed-method insights, future research should transition toward quasi-experimental designs to test targeted structural interventions. Evaluating the efficacy of specific platform adaptations—such as personalized content delivery, adaptive pacing, or AI-assisted feedback templates—could provide stronger evidence regarding causal effects and the effectiveness of targeted interventions.

## Data Availability

The raw data supporting the conclusions of this article will be made available by the authors, without undue reservation.

## Appendix B

**Table A1:** Measurement items.

Construct	Code	Items	Source
Information Overload (IO)	IO1	I am often distracted by too much information on the MOOC.	Chen et al. (2011)
	IO2	There is so much information on the MOOC every day that I need to check and process; I feel inundated.	
	IO3	The large amount of learning-related information on the MOOC sometimes makes me feel burdened when processing it.	
	IO4	The volume of information on the MOOC is huge, but only a small portion of it is necessary for me.	
System Feature Overload (SFO)	SFO1	There are many redundant features in the MOOC.	Karr-Wisniewski and Ying (2010)
	SFO2	The MOOC platform constantly adds many new features, but their effect is often poor.	
	SFO3	The MOOC includes many poorly performing features, with relatively few well-performing ones.	
	SFO4	Some feature settings on the MOOC feel complicated to operate.	
Social Overload (SO)	SO1	Following the status of instructors or peers on the MOOC costs me a lot of time.	Zhang et al. (2016)
	SO2	Processing questions/tasks assigned by the instructor on the MOOC costs me a lot of time.	
	SO3	Browsing the course status of instructors or students on the MOOC requires a lot of my time commitment.	
Expectation Disconfirmation (ED)	ED1	I encountered more problems when using the MOOC than I expected.	Bhattacherjee (2001)
	ED2	My experience using the MOOC was not as good as I had expected.	
	ED3	Overall, the benefits derived from using the MOOC were less than I had expected.	
Usage Fatigue (UF)	UF1	I sometimes feel weary/tired of the MOOC.	Ravindran et al. (2014); (Bhattacherjee (2001)
	UF2	I sometimes feel bored/annoyed by the MOOC.	
	UF3	Using the MOOC sometimes leaves me feeling exhausted.	
	UF4	I am not very concerned about the MOOC's dynamic notifications/reminders.	
Dissatisfaction (DIS)	DIS1	I am dissatisfied with the overall experience of using the MOOC.	Gu et al. (2021)
	DIS2	I am not delighted with the overall experience of using the MOOC.	
	DIS3	I am not content/satisfied with the overall experience of using the MOOC.	
	DIS4	I am not happy/joyful with the overall experience of using the MOOC.	
Discontinuous Usage Intention (DUI)	DUI1	I will reduce the frequency of my MOOC usage in the future.	Maier et al. (2015)
	DUI2	I will sometimes temporarily stop using the MOOC for a while before resuming.	
	DUI3	I might switch to using other learning platforms in the future.	
	DUI4	I might stop using the MOOC if possible.	

To enhance methodological transparency and address concerns regarding the high scale reliability, this appendix provides example items for each construct, demonstrating their context-specific adaptation for MOOCs. All items were measured on a 7-point Likert scale (1 = Strongly Disagree, 7 = Strongly agree).
